# Systematic Angle Random Walk Estimation of the Constant Rate Biased Ring Laser Gyro

**DOI:** 10.3390/s130302750

**Published:** 2013-02-27

**Authors:** Huapeng Yu, Wenqi Wu, Meiping Wu, Guohu Feng, Ming Hao

**Affiliations:** College of Mechatronics and Automation, National University of Defense Technology, Changsha 410073, Hunan, China; E-Mails: wenqiwu_lit@hotmail.com (W.W.); meipwu@hotmail.com (M.W.); guohu_feng@hotmail.com (G.F.); minghao_lit@hotmail.com (M.H.)

**Keywords:** rate biased technique, ring laser gyro, fast orthogonal search, Allan variance

## Abstract

An actual account of the angle random walk (ARW) coefficients of gyros in the constant rate biased rate ring laser gyro (RLG) inertial navigation system (INS) is very important in practical engineering applications. However, no reported experimental work has dealt with the issue of characterizing the ARW of the constant rate biased RLG in the INS. To avoid the need for high cost precise calibration tables and complex measuring set-ups, the objective of this study is to present a cost-effective experimental approach to characterize the ARW of the gyros in the constant rate biased RLG INS. In the system, turntable dynamics and other external noises would inevitably contaminate the measured RLG data, leading to the question of isolation of such disturbances. A practical observation model of the gyros in the constant rate biased RLG INS was discussed, and an experimental method based on the fast orthogonal search (FOS) for the practical observation model to separate ARW error from the RLG measured data was proposed. Validity of the FOS-based method was checked by estimating the ARW coefficients of the mechanically dithered RLG under stationary and turntable rotation conditions. By utilizing the FOS-based method, the average ARW coefficient of the constant rate biased RLG in the postulate system is estimated. The experimental results show that the FOS-based method can achieve high denoising ability. This method estimate the ARW coefficients of the constant rate biased RLG in the postulate system accurately. The FOS-based method does not need precise calibration table with high cost and complex measuring set-up, and Statistical results of the tests will provide us references in engineering application of the constant rate biased RLG INS.

## 1. Introduction

A ring laser gyro (RLG) is an ideal angular measurement sensor for a high precision inertial navigation system (INS) [1–4]. Several well-known firms in the inertial field have been working on laser gyro navigation systems employing the rate biased technique as a technical and economical solution for long endurance accurate autonomous navigation [1,2,5,6].

The rate biased technique requires only a single drive *versus* the three equivalent drives in the mechanically dithered technique and combines platform-like high dynamic stability with typical strapdown simplicity and robustness. To avoid the stringent requirement for rapid turntable reversal, a special constant rate mode of the rate biased RLG was developed, in which the RLG is operated through continuous turntable rotation above the lock-in threshold in a single direction [1].

All types of inertial sensors exhibit errors such as bias, scale factor, and noise, among others. As a rotation modulation system for the constant rate biased RLG INS, eliminating fixed or slowly changing bias errors of equivalent horizontal sensors through turntable rotation guarantees significantly higher navigation accuracy [1,2,7,8]. Besides, the bias and scale factor errors are deterministic errors that can be greatly eliminated by proper calibration [1,2]. Main terms contributing to the scale factor of the constant rate biased RLG were derived from theoretical and experimental study of the constant rate biased RLG in [9]. The mechanically dithered RLG exhibits an additional source of ARW error in typical RLG INS', but the rate biased RLG avoids this source of noise by replacing the vulnerable and noisy dithering mechanism [1,7]. However, angle random walk (ARW) error cannot be attenuated by the turntable rotation [7,8], making it the dominant RLG error source for the rate biased RLG INS.

With its potential for high measuring accuracy, it is valuable to study the operation performance of the constant rate biased RLG to best exploit the inherent quality of the RLG system. The most compelling evidence is the fact the way to improve operation performance of the inertial sensor is to know more details about the noise components in the sensor measured data [7]. As a directly measurable quantity, Allan variance (AVAR) is an effective way to determine the characteristics of various types of noise terms in the inertial sensor data [10].

An actual account of ARW coefficients of the gyros in the constant rate biased rate RLG INS is most important to determine the a prior probability for the Kalman filtering in initial alignment or integrated navigation and so on. However, there is still a dearth of effective experimental methods to characterize the constant rate biased RLG noise. To avoid the need for high cost precise calibration tables and complex measuring set-ups, estimating the ARW coefficients of the gyros in the constant rate biased RLG INS directly is attractive. Since ARW is a high frequency noise, high frequency data is acquired for proper determination of the ARW error using AVAR [11]. Furthermore, calibration accuracy on the system is limited due to turntable precision, so turntable dynamics and other external noises should be taken into consideration. Consequently, full rotation interval sample (FRIS) [12] is not applicable to this case because of the practical limitation of the turntable rotation rate.

This paper aims to develop a practical method for characterizing ARW error present in the constant rate biased RLG measured data. Since turntable dynamics and other external noises would corrupt AVAR calculation, we present an experimental method for separating them from the constant rate biased RLG measured data and verify the effectiveness of this method on a postulate system.

The remaining of this paper is organized as follows: Section 2 presents a brief description of the postulate system used as the experiment platform and an overview of the AVAR technique. In Section 3, an ideal observation model of the RLG triad mounted on a turntable is firstly discussed, and a method based on sampling the RLG measured angle at full rotation interval is then introduced. Next, problems of characterizing ARW of the constant rate biased RLG are addressed. To tackle these problems, a practical observation model is discussed, and an experimental method based on the fast orthogonal search (FOS) is proposed. Section 4 presents the validity check of the FOS-based method and the estimation results of the ARW coefficients for both the mechanically dithered RLG and the constant rate biased RLG. Conclusions are drawn in Section 5.

## 2. Background Work and Some Preliminaries

### 2.1. Experiment Platform Configuration and Definition of Coordinate Frames

**Figure 1.**
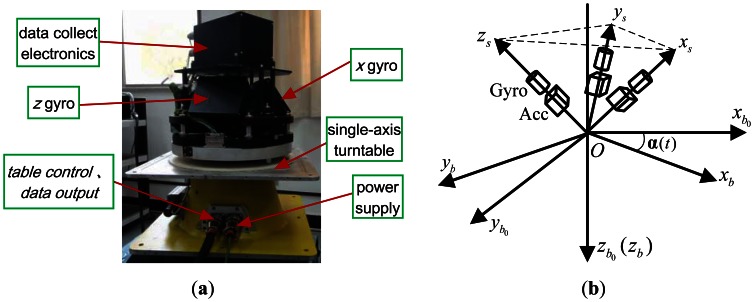
(**a**) The postulate system; (**b**) Coordinate frames in the postulate system.

To characterize the ARW of the RLG in the two operation modes and make comparisons between them, we set up a postulate system [13] as the experimental platform (see Figure 1(a)), the principal idea of which is close to that of WSN-5 [1] or INS PL41 MK4 [2]. The postulate system and coordinate frames in the postulate system are shown in Figure 1.

With angular divisions in the form of a line grid which is read out photoelectrically, divisions of up to 7,200,000 lines on the circle perimeter of the angle encoder are realized, corresponding to an angular resolution of 0.18”. Furthermore, when the system passes each full rotation, the photoelectric null indicator on the angle encoder permits one to determine the 360° angle with an accuracy of 2”.

Without loss of generality, the local-level frame *n* is selected as the navigation frame, with *x_n_* directing north, *y_n_* directing east and *z_n_* downwards vertically. We denote by *e* the Earth-Centered Earth-Fixed frame and by *i* some chosen inertial frame.

Ignoring the fact that errors due to precision limitations in manufacture and installation of the installation structure are extraordinarily small, we denote by *s* the IMU sensors' orthogonal frame where each axis lies along each of the RLG triad sensitive axes.

We denote by *b* the body frame, where *x_b_* lies along the projection of gyro sensitive axis *x_s_* on the turntable plane, *z_b_* lies along the turntable shaft center and downwards vertically to the turntable plane, and *y_b_* lies on the turntable plane to form an orthogonal frame. The *b* frame is rotated along with the turntable rotation. We define the *b*_0_ frame when axis *x_b_* coincides with the photoelectric null indicator.

The transformation matrix from the *b* frame to the *s* frame should be determined by laboratory calibration prior to practical use. Its general form can be described by the following expression:

(1) $${\text{C}}_{b}^{s}=\left[\begin{array}{ccc} \sqrt{1-e_{x}^{2}} & 0 & e_{x}\\ -e_{x}e_{y}/\sqrt{1-e_{x}^{2}} & e_{z}/\sqrt{1-e_{x}^{2}} & e_{y}\\ -e_{x}e_{z}/\sqrt{1-e_{x}^{2}} & -e_{y}/\sqrt{1-e_{x}^{2}} & e_{z} \end{array}\right]$$

where *e_x_*, *e_y_* and *e_z_* are the proportion of turntable shaft input angular rate imparted on the three gyros, namely $1/\sqrt{3}$ for each [1,2]. From statistics results of repetitive laboratory calibration done on the experimental platform, we obtain:

(2) $$\text{max}\left\{\left|\delta e_{x}\right|,\left|\delta e_{y}\right|,\left|\delta e_{z}\right|\right\}\leq 0.002$$

where δ*e_x_*, δ*e_y_* and δ*e_z_* are the deviations of *e_x_*, *e_y_* and *e_z_* from their ideal value $1/\sqrt{3}$, and the operator “max{}” represents the maximum of its calculated variables.

### 2.2. An Overview of AVAR Analysis

Many possible noise sources mentioned in [10,11] can be present in the measured data. However, in this study we only consider noise terms that are either known to exist in the RLG, or otherwise influence its measured data.

Considering two data records, AVAR of each data record at any given *τ*, denoted as *σ*_1_(*τ*) and *σ*_2_(*τ*), can be computed according to [11]. Then, AVAR of the difference between the two data records can be expressed as:

(3) $$\sigma_{\text{diff}}^{2}\left(\tau \right)=\sigma_{1}^{2}\left(\tau \right)+\sigma_{2}^{2}\left(\tau \right)=\frac{6{\bar{Q}}^{2}}{\tau^{2}}+\frac{2{\bar{N}}^{2}}{\tau }+\frac{4{\bar{B}}^{2}}{\pi }\ln2+\frac{2{\bar{K}}^{2}}{3}\tau +{\bar{R}}^{2}\tau^{2}$$

where *Q* is the quantization noise coefficient, *N* is the ARW coefficient, *B* is the bias instability coefficient, *K* is the rate random walk coefficient, *R* is the rate ramp coefficient, and *τ* is the time length of data clusters. The symbol “¯” represents the average value of noise coefficients of the two data records. In the least mean squares sense, the coefficients in Equation (3) can be determined within a given accuracy [10,11].

## 3. RLG Observation Model Development and Noise Separation Method

### 3.1. Ideal Observation Mode

Theoretically, angular rate vector as measured by the RLG triad on the experimental platform is given by:

(4) $$\omega_{is}^{s}={\text{C}}_{b}^{s}\left[\omega_{nb}^{b}+{\text{C}}_{b_{0}}^{b}{\text{C}}_{n}^{b_{0}}\left(\omega_{ie}^{n}+\omega_{en}^{n}\right)\right]$$

where $\omega_{is}^{s}$ is the RLG triad measured angular rate, $\omega_{ie}^{n}$ the turn rate of the Earth expressed in the *n* frame, $\omega_{en}^{n}$ the turn rate of the *n* frame with respect to the *e* frame, and $\omega_{nb}^{b}$ the turn rate of the *b* frame with respect to the *n* frame.

As the experiment platform is placed on a vibration insulating foundation in laboratory, which means that $\omega_{\text{en}}^{\text{n}}=0$, the following parameter may be defined:

(5) $$\eta ={\left[\begin{array}{ccc} \eta_{1} & \eta_{2} & \eta_{3} \end{array}\right]}^{\text{T}}={\text{C}}_{\text{n}}^{{\text{b}}_{0}}\omega_{\text{ie}}^{\text{n}}$$

where ***η*** is a constant value vector.

From the above equations, measured angle of each RLG is determined as the integral of $\omega_{is}^{s}$ from time 0 to time *t*, yields:

(6) $$\theta \left(t\right)=\left[\begin{array}{c} e_{x}\eta_{3}t+e_{x}\alpha +\frac{k_{x1}}{\Omega }\sin\alpha -\frac{k_{x2}}{\Omega }\left(\cos\alpha -1\right)\\ e_{y}\eta_{3}t+e_{y}\alpha +\frac{k_{y1}}{\Omega }\sin\alpha -\frac{k_{y2}}{\Omega }\left(\cos\alpha -1\right)\\ e_{z}\eta_{3}t+e_{z}\alpha +\frac{k_{z1}}{\Omega }\sin\alpha -\frac{k_{z2}}{\Omega }\left(\cos\alpha -1\right) \end{array}\right]$$

where $\theta (t)={\left[\begin{array}{ccc} \theta_{x}(t) & \theta_{y}(t) & \theta_{z}(t) \end{array}\right]}^{T}$ represent the RLG triad measured angles, *k_xi_*, *k_yi_*, *k_zi_* (*i* = 1, 2) the respective constant value coefficients, ***α*** the rotation angle of the axis *x_b_* with respect to the photoelectric null indicator, ***Ω*** the rotation rate of turntable shaft. We have:

(7) $$\alpha =\int_{0}^{t}\Omega dt$$

### 3.2. Problem Formulation

According to [12], isolation of turntable rotation periodic components from the RLG triad measured angle can be implemented by FRIS, which is to sample *_θ_*_(_*_t_*_)_ at the time the turntable shaft passes each full rotation. Assuming that turntable rotation angle is 2π from *t*_1_ to *t*_2_, from Equation (6) we can obtain:

(8) $${\theta \left(t\right)|}_{t_{1}}^{t_{2}}=\left[\begin{array}{ccc} {\theta_{x}\left(t\right)|}_{t_{1}}^{t_{2}} & {\theta_{y}\left(t\right)|}_{t_{1}}^{t_{2}} & {\theta_{z}\left(t\right)|}_{t_{1}}^{t_{2}} \end{array}\right]=\left(2\pi +\eta_{3}\left(t_{2}-t_{1}\right)\right){\left[\begin{array}{c} e_{x}\\ e_{y}\\ e_{z} \end{array}\right]}^{\text{T}}$$

Taking the difference between two components of the resultant difference data record $\theta (t){|}_{t_{1}}^{t_{2}}$, for example $\theta_{x}(t){|}_{t_{1}}^{t_{2}}$ and $\theta_{z}(t){|}_{t_{1}}^{t_{2}}$, gives:

(9) $${\delta \theta_{xz}|}_{t_{1}}^{t_{2}}={\theta_{x}\left(t\right)|}_{t_{1}}^{t_{2}}-{\theta_{z}\left(t\right)|}_{t_{1}}^{t_{2}}=\left(e_{x}-e_{z}\right)\left[2\pi +\eta_{3}\left(t_{2}-t_{1}\right)\right]$$

Considering that δ*e_x_*, δ*e_y_* and δ*e_z_* are rather small, which implies *e_x_*–*e_z_* ≈ 0, it is evident from Equation (9) that components simultaneously imparted on the two gyros will be removed effectively. Theoretically, the resultant difference data record $\delta \theta_{xz}{|}_{t_{1}}^{t_{2}}$ is composite of noise processes present in *x* and *z* gyros, and AVAR can be used to estimate the average ARW coefficient of *x* and *z* gyros.

**Figure 2.**
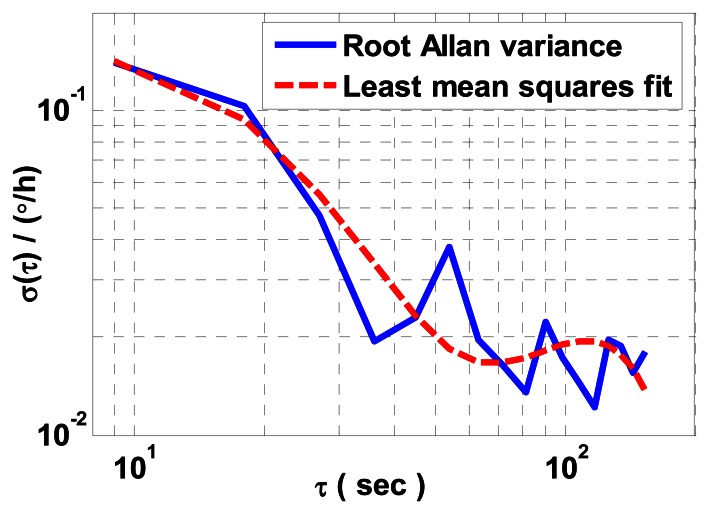
AVAR result of *x* and *z* gyros measured data processed by the FRIS method.

To verify its precision for characterizing ARW of the constant rate biased RLG, we process *x* and *z* gyro measured data collected over two hours on the experimental platform by utilizing the FRIS method. The log-log plot the *σ*(*τ*) *versus τ* within an estimation accuracy of 10% is shown in Figure 2.

Using least mean squares fit, a value of $0.0498{}^{\circ}/\sqrt{\text{h}}$ is obtained for the average ARW coefficient of *x* and *z* gyros. In view of the results of the theoretical analysis in [5] and the experimental tests in [2,6], the estimated average ARW coefficient here is dubious and incorrect. Therefore, two problems have to be addressed.

The first problem involves turntable dynamics and other external noises. In Figure 2, the AVAR shows sinusoidal behavior with successive peaks, which illustrates that the AVAR estimation is still contaminated by other periodic components which are not integer harmonics of either ***α*** or *t*.

**Figure 3.**
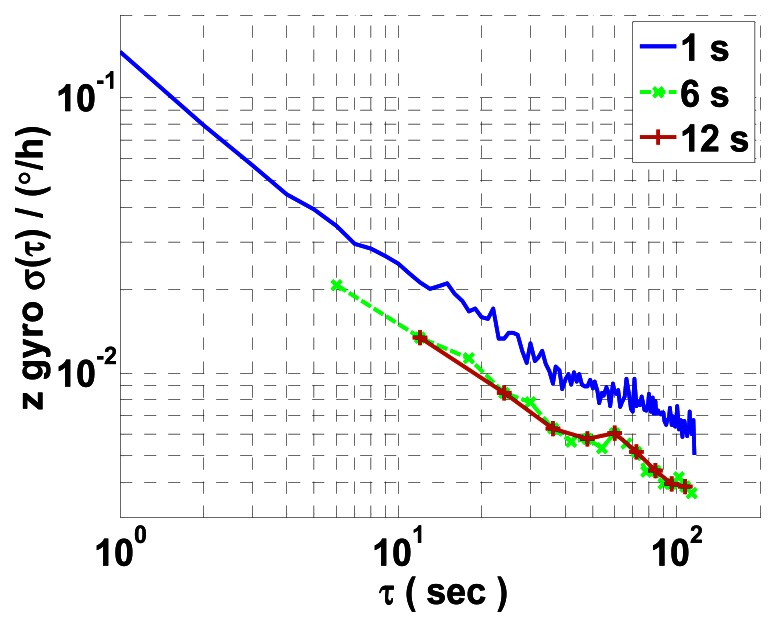
AVAR results of one measured data of *z* mechanically dithered RLG under stationary condition at different sample rates.

The second problem relates to the correlation time of the ARW error of RLG. An appropriate sample rate/data record length should be chosen to overlap about the correlation time of the ARW error [11] in AVAR calculation. To state this problem clearly, we also process one measured data of *z* mechanically dithered RLG collected for two hours on the experimental platform under stationary conditions at different sample rates. Figure 3 shows the log-log plots of *σ*(*τ*) *versus τ* with a maximum estimation percentage error of 10% at different sample rates.

Using least mean squares fit, the estimated ARW coefficients of *z* mechanically dithered RLG are $2.5006\times 10^{-4}{}^{\circ}/\sqrt{\text{h}}$, $3.3011\times 10^{-3}{}^{\circ}/\sqrt{\text{h}}$, $4.5305\times 10^{-3}{}^{\circ}/\sqrt{\text{h}}$ at 1 s, 6 s and 12 s sample time intervals, respectively. Compared with the results listed in Table 1, we believe that 1 s is an appropriate sample period for characterizing the ARW of RLG. The resultant difference data record should be acquired for a shorter FRIS period of time, which gives reason for the wrong result shown in Figure 2. However, because of the practical limits of the turntable, a higher rotation rate will result in lower angle encoder measuring precision and much more turntable noise. Therefore, the FRIS method is not applicable to this case.

### 3.3. Practical Observation Model and Noise Separation Method Based on the FOS

To tackle the problems formulated above, a practical observation model is discussed first. Then an experimental method based on the FOS is proposed to separate periodic components due to turntable motion and other external disturbances from the RLG measured angle in a two-step procedure. At last, the resultant gyro noise difference data record can be sampled at 1 s time interval to improve the estimation resolution of the ARW coefficient of RLG using AVAR.

Taking *x* gyro as an example, we consider first the effect imposed by turntable rotation. As was mentioned above, main errors introduced by the turntable rotation to *x* RLG measured angle, denoted as Δ*θ_x,rot_*(***α***), can be expressed in the form of *n*-order harmonics of ***α***:

(10) $$\Delta \theta_{x,\text{rot}}\left(\alpha \right)=\sum_{i=1}^{n}\left[k_{x\left(2+2i-1\right)}\sin\left(i\alpha \right)+k_{x\left(2+2i\right)}\left(\cos\left(i\alpha \right)-1\right)\right]$$

where *k_xi_* (*i* = 3,···, 2+2*n*) represents the respective constant value coefficient.

Other external periodic disturbances, denoted as Δ*θ_x,elec_*(*t*), also contribute to *x* RLG measured angle and should be modeled. We may write:

(11) $$\Delta \theta_{x,\text{elec}}\left(t\right)=\sum_{j=1}^{m}\left[l_{x(2j-1)}\sin\left(\omega_{j}t\right)+l_{x(2j)}\left(\cos\left(\omega_{j}t\right)-1\right)\right]$$

where *l_j_* (*j* = 1, ···, *m*) represents the respective constant value coefficient.

From Equations (6), (10) and (11), a practical observation model may be derived:

(12) $$\theta_{x}^{\prime }\left(t,\alpha \right)=\theta_{x}\left(t\right)+\Delta \theta_{x,\text{rot}}\left(\alpha \right)+\Delta \theta_{x,\text{elec}}\left(t\right)+n_{x}\left(t\right)$$

where $\theta_{x}(t,\alpha )$ represents the practical observation model in true environment, *n_x_* is the *x* gyro random error, which is the composite of gyro noise *n_x, t_* and random errors *n_x_*,_α_ caused by the turntable motion.

Due to the variation of turntable rotation rate, Δ*θ_x,rot_*(***α***) and Δ*θ_x,elec_*(*t*) should be described with different scales (***α*** or *t*), thus the traditional least mean squares fit technique cannot isolate the multiple errors. Rearranging Equation (12) in accordance with the scales (***α*** or *t*), this becomes:

(13) $$\theta_{x}^{\prime }\left(t,\alpha \right)=\theta_{x}^{\prime \prime }\left(t\right)+\theta_{x}^{\prime }\left(\alpha \right)$$

where:

(14) $$\theta_{x}^{\prime \prime }\left(t\right)=-\sum_{j=1}^{m}l_{x(2j)}+e_{x}\eta_{3}t+\sum_{j=1}^{m}\left[l_{x(2j-1)}\sin\left(\omega_{j}t\right)+l_{x(2j)}\cos\left(\omega_{j}t\right)\right]+n_{x,t}$$

(15) $$\theta_{x}^{\prime }\left(\alpha \right)=-\sum_{i=1}^{n}k_{x(2+2i)}^{\prime }+e_{x}\alpha +\sum_{i=1}^{n}[k_{x(2+2i-1)}^{\prime }\sin\left(i\alpha \right)+k_{x(2+2i)}^{\prime }\cos\left(i\alpha \right)]+n_{x,\alpha }$$

(16) $$\left\{ \begin{array}{cc} k_{x(2i+j)}^{\prime }=k_{x\left(2i+j\right)}+k_{xj} & i=1;j=1,2\\ k_{x(2i+j)}^{\prime }=k_{x\left(2i+j\right)} & i=2,\cdots ,n;j=1,2 \end{array}\right.$$

As can be seen in Equations (14) and (15), $\theta_{x}^{\prime \prime }(t)$ and $\theta_{x}^{'}(\alpha )$ have an analogous description form. This potentially provides us the possibility of approaching a unified algorithm to remove both of them, even though the scales in the two equations are not identical.

To describe Equations (14) and (15) in a unified form, we may write:

(17) $$\theta_{x}^{\prime }\left(\lambda \right)=\chi_{x0}+\chi_{x1}\lambda +\sum_{i=1}^{N}\left[\chi_{x(2i)}\sin\left(\omega_{i}\lambda \right)+\chi_{x(2i+1)}\cos\left(\omega_{i}\lambda \right)\right]+n_{x,\lambda }$$

where *λ* represents the scale in ***α*** or *t*, and χ*_xi_* (*i* = 0,···, 2*N*+1) can be determined by corresponding items in Equation (14) or (15). Noting that, when *λ*= *t*, *N* equals to *m* in Equation (14); when *λ*= ***α***, *N* equals to *n*, and ω*_i_* equals *i* in Equation (15). According to the analysis above, special attention should be paid to the following points:

1. Equations (14) and (15) are both nonlinear;
2. The RLG measured angle is sampled at a fixed time interval in the postulate system, however variation of turntable rotation rate causes ***α*** sampled at an unequal interval;
3. Frequencies of periodic components in Equation (14) or (15) may be very close, so traditional filtering techniques (*i.e.*, Fourier series analysis) may not characterize them precisely for frequency leakage and low signal to noise ratio.

The FOS has been illustrated for efficiently constructing accurate and parsimonious models of nonlinear dynamic systems (*i.e.*, sinusoidal series data) without requiring the data to be equally sampled [14–18]. Therefore, the FOS is a suitable solution for problems to be solved in this section.

We may write Equation (17) in a general form for the FOS:

(18) $$\theta_{x}^{\prime }\left(\lambda \right)=\sum_{i=0}^{2N+1}\chi_{xi}P_{i}\left(\lambda \right)+n_{x,\lambda }$$

where:

(19) $$\left\{ \begin{array}{cc} P_{i}\left(\lambda \right)=\lambda^{i} & i=0,1\\ P_{i}\left(\lambda \right)=\sin\left(\omega_{j}\lambda \right) & i=2j,j=1,\cdots N\\ P_{i}\left(\lambda \right)=\cos\left(\omega_{j}\lambda \right) & i=2j+1,j=1,\cdots N \end{array}\right.$$

By selecting candidates *P_i_*(*λ*) as pairs of sine and cosine terms at each of the frequencies chosen, constructing the sinusoidal series data is accomplished naturally. The magnitude and phase at the candidate frequency can be determined by the least mean squares fit. Further details concerning this algorithm can be found in [14–18]. To characterize ARW of the RLG triad mounted on a turntable effectively, an experimental method based on the FOS for isolating periodic components of $\theta_{x}^{\prime \prime }(t)$ and $\theta_{x}^{'}(\alpha )$ is proposed as follows:

Step 1. Remove periodic components of $\theta_{x}^{'}(\alpha )$ in Equation (15) from the RLG measured angle using the FOS;
Step 2. Remove periodic components of $\theta_{x}^{\prime \prime }(t)$ in Equation (14) from the resultant RLG measured angle through **Step 1**; after one RLG measured angle data record is processed by the above two-step procedure, main components left in the resultant RLG measured angle can be expressed as:
  (20) $$\theta_{x}^{\prime \prime }\left(t,\alpha \right)=e_{x}\left(\eta_{3}t+\alpha \right)-\left(\sum_{j=1}^{m}l_{2j}+\sum_{i=1}^{n}{k^{\prime }}_{x\left(2+2i\right)}\right)+n_{x,\alpha }+n_{x,t}$$
Step 3. Taking difference between the resultant data records of *x* and *z* RLG measured angles from **Step 2**, remainder components of turntable motion and the Earth rotation are isolated in succession. As was done in (9), we obtain:
  (21) $${\theta^{\prime \prime }}_{x,z}\left(t,\alpha \right)=\theta_{x}^{\prime \prime }\left(t,\alpha \right)-{\theta^{\prime \prime }}_{z}\left(t,\alpha \right)=n_{x,t}-n_{z,t}+\theta_{0}$$
  where *θ*_0_ is a constant value which can be easily eliminated:
  (22) $$\theta_{0}=\sum_{j=1}^{m}\left(l_{z(2j)}-l_{x(2j)}\right)+\sum_{i=1}^{n}\left({k^{\prime }}_{z\left(2+2i\right)}-{k^{\prime }}_{x\left(2+2i\right)}\right)$$
Step 4. Sample the resultant gyro noise difference data record $\theta_{x,z}^{\prime \prime }(t,\alpha )$ at 1 s time interval, and then estimate the average ARW coefficient of the two gyros by AVAR.

For convenience, this four-step method proposed above to characterize ARW of the RLG triad mounted on a turntable is called as the FOS-based method.

## 4. Experimental Results and Discussions

By characterizing ARW of the mechanically dithered RLG under stationary and turntable rotation conditions, validity of the FOS-based method is checked. Then, the average ARW coefficient of the constant rate biased RLG is estimated by utilizing the FOS-based method.

On the experimental platform, sample time of the RLG measured angle, denoted as *τ*_s_, is 2 ms. In the AVAR calculation, the unit of *σ*(*τ*) is °/h and the gyro noise data record is sampled at a time interval *τ*_0_ = 1 s. Taking the long-term stability of the gyros into account, required tests are done three times on different days under respective turntable rotation conditions. Laboratory tests are done by fixing the experimental platform on a vibration insulating foundation, without any temperature control device or other such instruments. Only *x* and *z* RLGs are available on the experiment platform.

### 4.1. Validity Check of the FOS-based Method

Let the RLG triad on the experiment platform operate in the mechanically dithered mode. The RLG triad measured angle is collected for two hours on the experimental platform when the turntable is stationary and the turntable rotates continuously in a single direction at 10°/s, 20°/s, 30°/s and 40°/s respectively.

**Figure 4.**
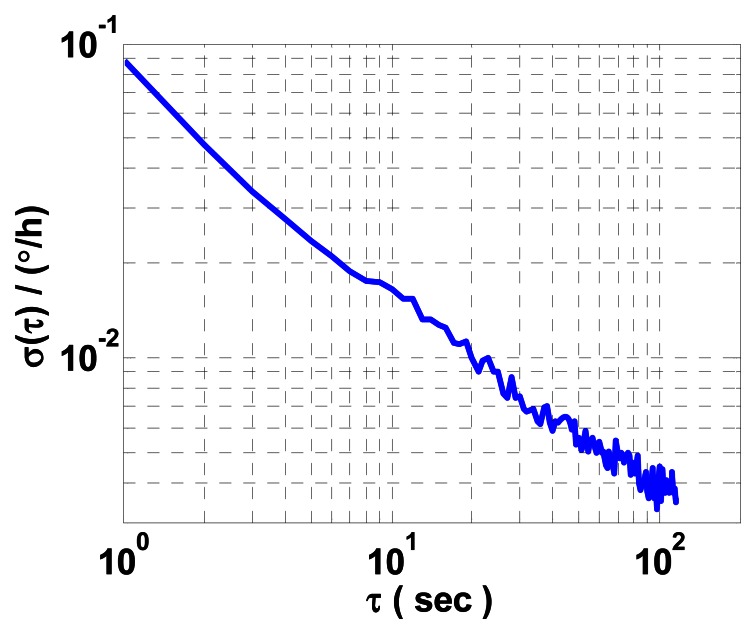
AVAR results of the resultant gyro noise difference data of *x* and *z* mechanically dithered RLGs under stationary condition.

**Figure 5.**
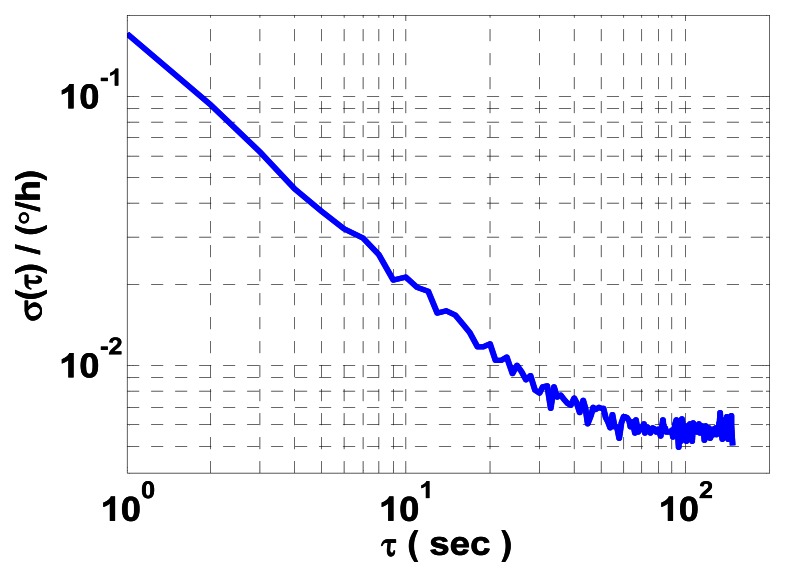
AVAR result of the resultant gyro noise difference data of *x* and *z* mechanically dithered RLGs when the turntable rotates at 40°/s.

(1) For data collected when the turntable is stationary, the ARW coefficient of the mechanically dithered RLG can be estimated after simple post-processing of the measured data with sampling the data records at a time interval *τ*_0_ = 1 s. To provide a reference for the FOS-based method, the average ARW coefficient of *x* and *z* mechanically dithered RLGs is estimated by AVAR after taking difference between the two measured data. Figure 4 shows a log-log plot of *σ*(*τ*) *versus τ* of the resultant gyro noise difference data under stationary condition, with an AVAR maximum estimation percentage error of 10%.

(2) For data collected under turntable rotation, the average ARW coefficient of *x* and *z* mechanically dithered RLGs is estimated by utilizing the FOS-based method. A log-log plot of *σ*(*τ*) of the resultant gyro noise difference data of *x* and *z* mechanically dithered RLGs *versus τ* when the turntable rotates at 40°/s is shown in Figure 5, with a maximum estimation percentage error of 10%.

Estimation results of the ARW coefficient of the mechanically dithered RLG under different turntable rotation conditions are listed in Table 1.

Comparing Figure 5 with Figure 2, various periodic components found in the RLG measured angle can be accurately removed by the FOS-based method, which is profitable to improve the least mean squares fit in AVAR calculation. In Table 1, it is illustrated that the FOS-based method can estimate the average ARW coefficient of *x* and *z* mechanically dithered RLGs accurately. In general, the FOS-based method is validated to be effective for characterizing ARW of the constant rate biased RLG mounted on a turntable.

### 4.2. Characterization of ARW of the Constant Rate Biased RLG

**Figure 6.**
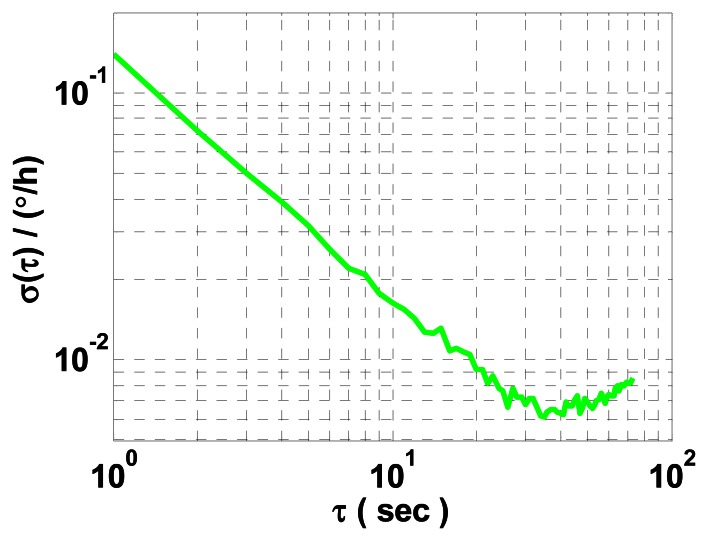
AVAR result of the resultant gyro noise difference data of *x* and *z* constant rate biased RLGs when the turntable rotates at 40°/s.

Let the RLG triad on the experimental platform operate in the constant rate biased mode, and turntable rotates continuously in a single direction at different turntable rotation rate, such as 10°/s, 20°/s, 40°/s, *etc.* By utilizing the FOS-based method, the average ARW coefficients of *x* and *z* constant rate biased RLGs are estimated. Figure 6 shows a log-log plot of *σ*(*τ*) *versus τ* of *x* and *z* constant rate biased RLGs, with an AVAR estimation maximum percentage error of 10%. Estimation results of the average ARW coefficient of the constant rate biased RLG are listed in Table 2.

Comparing Figure 6 with Figure 2, estimation accuracy of the average ARW coefficient of *x* and *z* constant rate biased RLGs is significantly improved. In Table 2, it should be noted that the average ARW coefficient approaches stability when the turntable rotates at a much higher rate, which may be attributed to scale factor nonlinearity at low turntable rotation rates [9]. Therefore, the results in Table 2 will provide us a reference to determine the turntable rotation rate optimally in practical engineering applications of the constant rate biased RLG INS. In Tables 1 and 2, it can also be concluded that the average ARW coefficient of RLG operating in the constant rate biased mode is one order less than that in the mechanically dithered mode. Using the statistics of the estimated results listed in Table 2, we can determine the *a priori* probability of gyro errors for the Kalman filtering or the actual precision limit of the constant rate biased RLG INS in initial alignment or integrated navigation and so on.

Taken together, the FOS-based method has been experimentally verified to be effective in characterization of the ARW of the RLG triad mounted on a turntable in the postulate system. In addition, statistic of our experimental tests have been used in the first laboratory tests of initial alignment on the experiment platform and achieved high initial alignment precision presented in [19].

## 5. Conclusions

An actual account of ARW coefficients of the gyros in the constant rate biased rate RLG INS is most important to determine the *a priori* probability for the Kalman filtering in initial alignment or integrated navigation applications and so on. Estimating the ARW coefficients of the gyros in the constant rate biased RLG INS directly will avoid the need for high cost precise calibration tables and complex measuring set-ups. However, estimation accuracy of this method is easily affected by turntable dynamics and other external noises.

A practical observation model of the RLG triad for denoising and increasing sample rate in AVAR calculation, and an experimental method based on the FOS was proposed to extract gyro noises from the RLG measured data were discussed. Validity of the FOS-based method was experimentally checked among the mechanically dithered RLG data collected on the experiment platform under stationary and turntable rotation conditions.

The experimental results show that the FOS-based method can estimate the ARW coefficients of the mechanically dithered RLG and the constant rate biased RLG accurately. Statistical results of the tests will provide us references in many aspects as mentioned above. Therefore, the FOS-based method is algorithmically simple and possessed of low cost attribute, and it will be greatly helpful in engineering application of the constant rate biased RLG INS.

## Figures and Tables

**Table 1.** Estimation results of the ARW coefficient of the mechanically dithered RLG under different turntable rotation conditions.

**Data Time**	**0°/s**	**10°s**	**20°/s**	**30°/s**	**40°/s**
1	3.6427	3.3903	4.2517	3.4121	3.4328
2	3.3709	3.4392	3.1152	3.2906	6.5700
3	3.7997	3.0869	3.6555	4.1709	4.5071

(a) Unit of the results is $10^{-4}{}^{\circ}/\sqrt{\text{h}}.$

**Table 2.** Estimation results of the average ARW coefficient of *x* and *z* constant rate biased RLGs by utilizing the FOS-based method.

**Data Time**	**10°/s**	**20°/s**	**40°/s**	**60°/s**	**80°/s**
1	8.3297	6.7278	7.4707	2.1993	3.4774
2	4.6176	2.6117	2.3985	2.9396	2.3414
3	6.8846	3.2470	2.3238	2.5313	2.6234

(a) Unit of the results is $10^{-5}{}^{\circ}/\sqrt{\text{h}}.$
